# Evaluation of **Mean Platelet Volume values in **lean women with polycystic ovary syndrome

**DOI:** 10.12669/pjms.303.4475

**Published:** 2014

**Authors:** Dilek Benk Silfeler, Raziye Keskin Kurt, Erhan Yengil, Burak Un, Secil Arica, Ali Baloglu

**Affiliations:** 1 Dilek Benk Silfeler, Department of Obstetrics and Gynecology, Faculty of Medicine, Mustafa Kemal University, Antakya, Hatay, Turkey.; 2 Raziye Keskin Kurt, Department of Obstetrics and Gynecology, Faculty of Medicine, Mustafa Kemal University, Antakya, Hatay, Turkey.; 3 Erhan Yengil, Department of Family Medicine, Faculty of Medicine, Mustafa Kemal University, Antakya, Hatay, Turkey.; 4 Burak Un, Department of Obstetrics and Gynecology, Faculty of Medicine, Mustafa Kemal University, Antakya, Hatay, Turkey.; 5 Secil Arica, Department of Family Medicine, Faculty of Medicine, Mustafa Kemal University, Antakya, Hatay, Turkey.; 6 Ali Baloglu, Department of Obstetrics and Gynecology, Faculty of Medicine, Mustafa Kemal University, Antakya, Hatay, Turkey.

**Keywords:** Mean Platelet Volume, Polycystic Ovary Syndrome, Lean

## Abstract

***Objective: ***Mean Platelet Volume (MPV) is an important indicator of platelet activation. It is known that MPV increases in patients with coronory artery disease, diabetes mellitus, atherosclerosis and Polycystic ovary syndrome (PCOS). Our aim was to measure the MPV in lean patients with polycystic ovary syndrome.

***Methods:*** The present study was designed to examine the platelet function by measuring MPV in non-obese women with PCOS. A total of 50 outpatients with PCOS were included. The control group consisted of 50 healthy subjects. Serum platelet, MPV, and white blood cell (WBC) levels were compared and evaluated retrospectively in all participants. These values were compared by statistical analysis.

***Results:*** There were no statistically significant difference in between groups regarding MPV (p═0.357), WBC (p═0,414) and platelet (p═0,666).

***Conclusion:*** There are studies implying MPV increase in PCOS patients, in our patients MPV levels did not correlate with PCOS except for patients with obesity. We think that PCOS itself has no effect on MPV levels and obesity changes MPV levels.

## INTRODUCTION

Polycystic ovary syndrome (PCOS) is the most common endocrinopathy seen in women of reproductive age with a ratio of 10%. The main characteristic features of the syndrome include chronic anovulation, hyperandrogenism and insulin resistance. Symptoms include hirsitusmus, acne vulgaris, anovulation and infertility.^1^^,^^2^

PCOS is an endocrinological and metabolic disorder in which a well known association with diabetes mellitus (DM) and coronory artery disease (CAD) exists in long term. It is thought that insulin resistance, dyslipidemia and hyperandrogenemia seen in PCOS increase the risk of coronory artery disease.^3^^-^^5^

Platelets play an important role in coagulation. Platelet size is related with platelet function and activation. Large sized platelets have greater hemoastatic functions therefore Mean Platelet Volume (MPV) is an important indicator of platelet activation. It is reported that MPV increases in CAD, DM, atherosclerosis and PCOS.^1^^,^^6^^-^^9^ There are some studies which examine the relation between PCOS and MPV in literature. In these studies it is reported that increased risk of CAD and atherosclerosis in PCOS is associated with MPV values. These studies included both obese and lean patients with PCOS. It is known that obesity is an independent risk factor for CAD.^6^^-^^9^

There was no record of MPV levels in non-obese PCOS patients in literature. The aim of the current study is to examine MPV values between lean women with PCOS and healthy women with regular menses.

## METHODS

It was a cross-sectional study. The study population consisted of 50 patients with PCOS and 50 healthy participants as a control group. We selected lean patients who visited gynecology outpatient clinic of Mustafa Kemal University Hospital from January 2010 to December 2012. PCOS was diagnosed according to Rotterdam group criteria in which patients must have at least two of three features (oligo or amenorrhea, clinical and/or biochemical signs of hyperandrogenism, ultrasonographic appearance of the polycystic ovary) after excluding other etiologies including hyperprolactinemia and non-classical adrenal hyperplasia^10^. Body mass index (BMI) was calculated as weight divided by the square of the height (kg/m2). Lean patients were defined to have a BMI from 18.5 to 24.9 kg/m2. Patients with hypertension, hyperprolactinemia, DM, thyroid diseases, BMI of >25 kg/m2.and other systemic diseases were excluded. The study was approved by the local ethical committee of Mustafa Kemal University. Patient information was obtained from the hospital records. BMI, complete blood count parameters, early follicular phase hormone profile, tyriglyceride, high-density lipoprotein (HDL), low-density lipoprotein (LDL), very low density lipoprotein (VLDL), blood glucose, insulin level, hemoglobin a1c (HbA1C) were recorded. Insulin resistance was calculated according to Homeostatic model assessment of insulin resistance (HOMA-IR) formula[HOMA-IR=fasting insulin (mIU/mL) x fasting glucose (mg/dL)/18)/22.5]. HOMA-IR were recorded Hormon profile included thyroid stimulating hormone (TSH), follicle stimulating hormone(FSH), luteinizing hormone (LH), prolactin (PRL), estradiol (E2), free testosterone, total testosterone, and rostenedione (AS), 17-hydroxyprogesterone (17-OHP), dehydroepiandrosterone sulphate (DHEAS) and sex hormone binding-globulin (SHBG). These were measured. All these measurements were detected in order to exclude chronic diseases other from PCOS and to choose the suitable participants.

Using Standard enzymatic methods with a fully automated random Access chemiluminescence-enhancedenzy meimmunoassay system (Roche Laboratory Systems, Mannheim, Germany). Plasma glucose level was determined by the glucose oxidase method. Plasma insulin was measured by electrochemiluminescence immunoassay (roche DİAGNOSTİC GmBH, Mannheim). Plasma lipid profile was analyzed by LX-20 Pro chemistry analyzers (Beckman Coulter). Complete blood count was performed by a CELL-DYN 3700 SL analyzer (Abbott Diagnostics, Chicago, Illinois, USA). Analysis was measured in duplicate in a random cohort of 20 participants Intra-assay coefficient of variability variation was 2% for MPV and 4% for platelet count.

SPSS v.13.0 program was used for statistical analysis of the study. Normal distribution of continuous variables were tested with Kolmogorov-Smirnov test. Student’s t test and Mann-Whitney U test were used for the comparison in between the groups. Pearson correlation analyses were used to evaluate relation between continuous variables. Lineer regression analysis was performed for statistically significant data in correlation test. All the results were considered statistically significant with a p value of <0.05.

**Table-I:** Demographic features, hematologic findings and lipid profiles of the groups (means±SD).

	*PCOS*	*CONTROL*	*P*
Age (years)	21.28±3.63	22.86±4.74	0.051*
FPG(mg/dl)	87.74±6.82	87.08±10.08	0.702**
HbA1C	4.95±0.54	5.08±0.55	0.262**
Menars Age(years)	13.39±1.36	12.7±2.25	0.093*
BMI (kg/m2)	19.71±1.68	19.79±1.62	0.812**
WBC	7.72±2.13	8.08±2.27	0.414**
Plt (x103/l)	275.86±84.28	282.28±62.21	0.666**
MPV (fL)	7.93±1.16	8.15±1.23	0.357**
Total cholesterol (mg/dl)	159.39±28.57	156.08±24.96	0.539**
LDL-C (mg/dl)	95.12±21.54	92.09±21.43	0.483**
HDL-C (mg/dl)	48.96±11.40	49.41±9.19	0.826**
Triglyceride (mg/dl)	76.00±28.75	72.69±28.52	0.566**

BMI, body-mass index; Htc, hematocrit; Plt, platelet; MPV, mean platelet volume;

LDL-C, low-density lipoprotein cholesterol; HDL-C, high-density lipoprotein cholesterol

* Mann-Whitney U test,

** Students’s t test

**Table-II:** Hormonal parameters of groups (means ± SD).

	*PCOS*	*CONTROL*	*p*
T.Testest (ng/dl)	2,28±1.50	0.59±0.23	0.600*
Prolactin (ng/ml)	19.87±11.41	16.01±5.00	0.096*
TSH (μIU/ml)	1.52±0.82	1.48±0.73	0.818**
LH (mIU/ml)	7.01±5.46	5.44±4.43	0.003*
FSH (mIU/ml)	5.03±1.54	5.91±1.76	0.010**
Estradiol (pg/ml)	38.87±20.82	43.28±28.35	0.378**
Insuline	9.79±9.02	9.19±3.82	0.383*
Homo IR	2.19±2.43	1.99±0.97	0.627*

* Mann-Whitney U test,

** Students’s t test.

## RESULTS

Dermographic features, hemalogical parameters and lipid profiles of participants are shown in Table-I. The mean age of PCOS patient group and control group were 21.28±3.63 and 22.86±4.74 respectively. No significant difference was observed between the groups of age and first menstrual age (p═0.051 vs. p═0.093). There were no statistically significant differences between groups regarding MPV, WBC, fasting blood glucose level and lipid profile. Comparison of groups for hormonal parameters were shown in Table-II.

PLT values were 275±84 (×10^3^/μL) in PCOS group and 282±62 (×10^3^/μL) in control group (p═0.666). PLT values were associated with WBC, MPV ve E2 values in PCOS group.As E2 ve WBC values increased, platalet values increased too (respectively p=0.031/r=0.305, p=0.003/r=0.406); platelet values were decreased as MPV values increased (p═0.004/r=-0.402) in control group, negative correlation was seen in between platelet and MPV values (p═0.000./r = -0.549).

MPV values were 7.93±1.16 (min:6-max:11.5) and 8.15±1.23 (min:5.7, max:11.6) in PCOS group and control group respectively. No statistical significant difference was observed between two groups (Table-I) (p═0.357).

## DISCUSSION

In this study; comparison of lean patients with PCOS group and control group revealed no significant difference in terms of MPV. MPV is a potential marker of platelet function. Larger platelets have more prothrombic activity. In recent years, it was reported that MPV is associated with coronary artery disease, HT, athereosclerosis and diabetes mellitus.^7^^-^^9^ In patients with PCOS there is a tendency for thrombosis.^10^ This hypercoogulation can be related to increased levels of WBC and MPV. Some studies have shown a relation between MPV and PCOS.^11^^,^^12^

In Slopien et al. study they found no difference in glucose and insulin levels between obese PCOS patients and their BMI and age matched control group but only test ester on and LH levels were higher in PCOS group.^12^ In Kebapcilar’s research, WBC, HOMA IR, fasting glucose level, insulin and MPV levels were significantly higher in PCOS group.^11^ In our study we observed no significant difference in glucose, insulin, WBC, HOMA IR and MPV levels between groups so we assume that the difference observed in Kebapcilar’s study might be due to obese patients with PCOS.

In Kebapcilar’s study, treatment of PCOS with etinilestradiol + siproteron asetat and metformin revealed a decrease in MPV levels. In addition to that there were significant decreases in BMI after treatment. Our hypothesis is that decreasing BMI actually changed the MPV level in this study. In our study we found no relation between increased MPV levels and patients with PCOS , excluding obese patients with PCOS might explain this result.

In Kebapcilar’s study MPV and WBC which imply the risk of atherosclerosis and CAD were found increased in PCOS patients.^11^ Orio et al. found higher WBC levels in PCOS.^13^ WBC levels are independent risk factor and prognostic indicator in development of CAD.^14^ In our study WBC levels were similar between groups and we think that excluding obese patients with PCOS might explain this result. Coban et al’s study demonstrated that MPV levels were increased in obese patients. This study support our results.

In conclusion it is known that there is a relation between PCOS and CAD, HT, DM, Atherosclerosis. MPV increases in CAD, DM, atherosclerosis. MPV levels are increased in obesity. Although there are studies implying MPV increase in PCOS patients, in this study we found no increase in MPV levels in patients with PCOS by excluding obese patients. We think that PCOS itself has no effect on MPV levels and it is obesity which changes the MPV levels.

## Authors Contribution:

***Dilek Benk Silfeler:*** Designed the research protocol and literature search.

***Raziye Keskin Kurt, Burak Un, Erhan Yengil: ***Conducted the study and did data analysis.

***Dilek Benk Silfeler, Ali Baloglu:*** Prepared the final draft for publication.
